# Novel, sensitivity-based antibiotic regimen for multidrug-resistant *Mycobacteriumabscessus* infection following cosmetic surgery

**DOI:** 10.1016/j.jdcr.2023.02.029

**Published:** 2023-04-11

**Authors:** Marlee Hill, Amanda S. Weissman, Michael Franzetti, Pamela Allen

**Affiliations:** University of Oklahoma Health Sciences Center, Department of Dermatology, Oklahoma City, Oklahoma

**Keywords:** mycobacterium abscessus, M. abscessus, multidrug-resistant, skin and soft tissue infection, antibiotics, novel, infection, gluteal augmentation with autologous fat transfer, complex antibiotic regimens, rapidly growing nontuberculous mycobacteria, RG- NTM, cosmetic surgery, post-surgical infection, medical tourism, SSTIs

## Introduction

Rapidly growing nontuberculous mycobacteria (RG-NTM) are a relatively uncommon, yet clinically significant, cause of surgical-site skin and soft tissue infections (SSTIs) following cosmetic procedures, classically those performed outside the United States. The increasing popularity of cosmetic tourism comes with associated challenges, as infections often present with diagnostic delays and treatment inadequacy secondary to nonspecific clinical findings and multidrug resistance. Such infections cause great morbidity as they typically require prolonged and complex antibiotic regimens, costly hospital stays, and surgical intervention. Mycobacterium abscessus complex is a particularly virulent and antimicrobial resistant species of RG-NTM found contaminating soil, water, or improperly sterilized medical instruments and can cause pulmonary and cutaneous infections.^1^ This report exemplifies a case of postsurgical, multidrug-resistant (MDR) *Mycobacterium abscessus* infection following gluteal augmentation with autologous fat transfer performed in the Dominican Republic (DR) with specific considerations regarding antimicrobial selection.

## Case report

A 33-year-old Black woman with a medical history of cosmetic surgeries performed in the DR complicated by MDR bacterial SSTIs presented to dermatology with a 6-month history of treatment-resistant cystic buttocks lesions.

In July 2021, the patient underwent a breast reduction and abdominoplasty performed in the DR. She developed wound dehiscence 3 weeks later causing an ampicillin-susceptible *Enterococcus* spp. and extended spectrum beta-lactamase *Klebsiella pneumoniae* SSTI susceptible only to amikacin, meropenem, and trimethoprim/sulfamethoxazole (TMP/SMX). She reported treatment with a 1-month intravenous (IV) course and then 3-month oral course of unknown antibiotics due to management at an outside facility. Then, in November 2021, she returned to the DR for a bilateral breast implant and gluteal augmentation with autologous fat transfer. In February 2022, she presented to an outside facility for a left buttock cystic lesion. The lesion was not cultured but treated with incision and drainage, a course of oral antibiotics, and blood cultures were negative. The cyst recurred 2 weeks later, and the patient was prescribed a different oral antibiotic without improvement.

**Fig 1:**
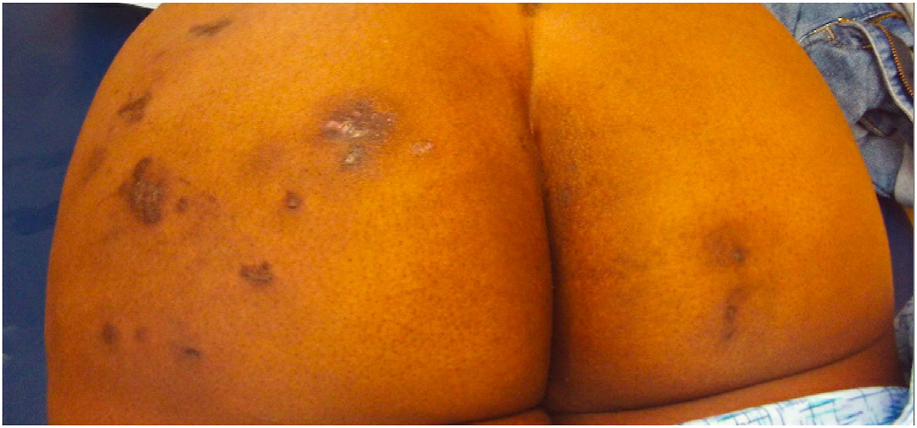
*Mycobacterium abscessus* surgical-site skin and soft tissue infection. Multiple indurated hyperpigmented to violaceous nodular plaques and one open nodule with a whitish-yellow hue on the mid-to-lateral left buttock with a few similarly appearing lesions on the right buttock.

Upon presentation to the dermatology clinic in August 2022, the patient recalled trialing doxycycline, levofloxacin, amoxicillin, clindamycin, minocycline, TMP/SMX, cephalexin, and rifampin with worsening progression of eight to nine painful, purulent cystic lesions. Physical examination of the lesions demonstrated indurated hyperpigmented to violaceous nodular plaques and one whitish-yellow scarring nodule on the left buttock (Fig 1). Punch biopsy of a left buttock lesion demonstrated an auramine-rhodamine, acid-fast, and Grocott methenamine silver negative-staining ulcer with underlying granulomatous inflammation. Fungal and bacterial cultures obtained 2 weeks later from a new nodular, left buttock lesion yielded MDR *M. abscessus* (Table I). Magnetic resonance imaging of the buttocks demonstrated small areas of fat necrosis or injection granuloma from medication administration without definite soft tissue abscess.

**Table I:** *Mycobacterium abscessus* susceptibility results in the patient

Antibiotic	MIC dilution	MIC interpretation
Amikacin	32	Intermediate
Cefoxitin	32	Intermediate
Ciprofloxacin	>8	Resistant
Clarithromycin	16	Resistant
Doxycycline	<16	Resistant
Imipenem	16	Resistant
Linezolid	16	Intermediate
Moxifloxacin	>8	Intermediate
Tigecycline	0.5	Resistant
TMP/SMX	>8	Resistant

*MIC*, minimum inhibitory concentration.

Due to extensive antimicrobial resistance, the infectious disease department and the Center for Disease Control and Prevention were consulted regarding appropriate treatment. Although not recommended at the time of diagnosis, surgical intervention remains under consideration to optimize infection control if necessary. The patient received 2 days of inpatient administration of IV amikacin, tigecycline, imipenem/cilastatin, and oral linezolid. She was discharged with a 6-month to 1-year outpatient regimen including IV amikacin and imipenem, as well as oral tedizolid and omadacycline. Clinical improvement of the lesions was noticed about 1 month after the start of the treatment. After 3 months, she is tolerating the medications with minimal side effects of tinnitus and yellowish teeth discoloration. The patient gave consent for their photographs and medical information to be published in print and online and with the understanding that this information may be made publicly available. Consent forms are available upon request.

## Discussion

Prompt antibiotic initiation, antimicrobial susceptibility testing, and surgical evaluation are crucial in preventing disease progression and worsening cosmesis in *M. abscessus* SSTIs. Clinical presentation of recurring violaceous nodules and subcutaneous abscesses, intractable to otherwise appropriate antibiotic regimens and debridement, should raise suspicion for RG-NTM infections in patients with a relevant surgical history. When biopsy results are non-revealing and clinical suspicion remains high, appropriate wound cultures should be obtained.^2^

In the case of this patient, a recent history of *Enterococcus* spp. and extended spectrum beta-lactamase *K. pneumoniae* SSTIs may have complicated her current treatment regimen. Her prior antibiotic treatment of likely MDR bacteria, plus the outside facility’s antibiotic regimen for the current *M. abscessus* infection, without wound cultures, likely played a role in the pan-resistant susceptibility results subsequently obtained. In cases where MDR organisms are suspected, appropriate cultures with organism identification and susceptibility results are essential prior to starting treatment to avoid further antibiotic resistance.

Evaluation of resistance patterns regarding isolates from the DR remains difficult as published and detailed literature is scarce. Despite the use of susceptibility testing, many antibiotic regimens continue to include isolate-resistant drug combinations. Similar susceptibility results, despite intermediate resistance to clarithromycin and imipenem, were demonstrated in an outbreak of *M. abscessus* infections following the abdominoplasties performed in the DR with the majority of cases treated with a macrolide plus cefoxitin, imipenem, amikacin, and/or linezolid.^3^ Similar reports of *M. abscessus* infections secondary to abdominoplasties in the DR and Caribbean Islands demonstrated sustained susceptibility to IV amikacin.^4^^,^^5^ Of the published, relevant cases, few show intermediate or complete resistance to all tested antimicrobials or describe regimens including tedizolid or omadacycline.

Innate resistance mechanisms including decreased cell envelope permeability, multidrug export systems, and antibiotic-target-modifying enzymes, in addition to an inducible erm(41) gene, enables *M. abscessus* resistance to a wide range of antibiotics.^6^ This patient’s treatment regimen is unique in that despite demonstrating doxycycline resistance and intermediate linezolid resistance, it includes novel, less commonly used antimicrobials of the same drug classes—omadacycline and tedizolid, respectively.

Omadacycline, a novel aminomethycycline, demonstrates a broad spectrum of *in vitro* and *in vivo* antimicrobial activity, in addition to remaining effective against the standard tetracycline resistance mechanisms of ribosomal protection and drug efflux pumps.^7^^,^^8^ Recent literature regarding tedizolid, a novel oxazolidinone, has demonstrated increased *in vitro* potency and effectiveness against linezolid-resistant bacterial strains, while demonstrating higher bioavailability, longer half-lives and more favorable side-effect profiling.^9^

Due to the growing popularity of medical tourism and increasing incidence of postsurgical infections, clinicians in the United States should have a high index of suspicion and lower threshold for considering RG-NTM SSTIs. Newer drug derivatives with high *in vitro/in vivo* activity and ability to evade typical resistance mechanisms should be considered to aid in the treatment of MDR isolates, with potential to increase treatment efficacy and decrease duration.

## Conflicts of interest

None declared.
